# Effect of digital ocular massage on intraocular pressure and Schlemm’s canal dimensions

**DOI:** 10.1038/s41598-024-56748-1

**Published:** 2024-03-13

**Authors:** Tiffany H. Y. Wu, Henry K. C. Lau, Carmelo H. Y. Lai, Ruby W. L. Wong, Antonia K. W. Wong, Andrew Kwok-cheung Lam

**Affiliations:** 1 School of Optometry, The Hong Kong Polytechnic University, Hung Hom, Kowloon, Hong Kong; 2 Research Centre for SHARP Vision, The Hong Kong Polytechnic University, Hung Hom, Kowloon, Hong Kong; 3 Centre for Eye and Vision Research, 17W Hong Kong Science Park, Science Park, Hong Kong

**Keywords:** Ocular massage, Schlemm’s canal, Trabecular meshwork, Intraocular pressure, Aqueous humor, Glaucoma, Risk factors

## Abstract

Digital ocular massage has been reported to temporarily lower intraocular pressure (IOP). This could be related to an enhanced aqueous humor outflow; however, the mechanism is not clearly understood. Using anterior segment optical coherence tomography, the Schlemm’s canal (SC) and trabecular meshwork (TM) can be imaged and measured. Here, 66 healthy adults underwent digital ocular massage for 10 min in their right eyes. The IOP and dimensions of the SC and TM were measured before and after ocular massage. All subjects demonstrated IOP reduction from 15.7 ± 2.5 mmHg at baseline to 9.6 ± 2.2 mmHg immediately after, and median of 11.6 mmHg 5-min after ocular massage (Friedman’s test, *p* < 0.001). There was significant change in SC area (median 10,063.5 μm^2^ at baseline to median 10,151.0 μm^2^ after ocular massage, Wilcoxon test, *p* = 0.02), and TM thickness (median 149.8 μm at baseline to 144.6 ± 25.3 μm after ocular massage, Wilcoxon test, *p* = 0.036). One-third of the subjects demonstrated collapse of the SC area (−2 to −52%), while two-thirds showed expansion of the SC area (2 to 168%). There were no significant changes in SC diameter (270.4 ± 84.1 μm vs. 276.5 ± 68.7 μm, paired *t*-test, *p* = 0.499), and TM width (733.3 ± 110.1 μm vs. 733.5 ± 111.6 μm, paired *t*-test, *p* = 0.988). Eyes with a higher baseline IOP demonstrated a greater IOP reduction (Pearson correlation coefficient *r* = −0.521, *p* < 0.001). Eyes with smaller SC area at baseline showed greater SC area expansion (Pearson correlation coefficient = −0.389, *p* < 0.001). Greater IOP reduction appeared in eyes with greater SC area expansion (Pearson correlation coefficient *r* = −0.306, *p* = 0.01). Association between change in IOP and change in TM thickness was not significant (Spearman’s *ρ* = 0.015, *p* = 0.902). Simple digital ocular massage is an effective method to lower IOP values, and change in the SC area was significantly associated with IOP changes.

## Introduction

Ocular massage has been reported in the literature to lower intraocular pressure (IOP) in some ocular conditions. This includes controlling IOP after trabeculectomy.^1,2^ The enlargement of filtration blebs as a result of ocular massage to improve aqueous humor outflow could be the mechanism by which IOP is reduced.^3^ Ocular massage also provided immediate IOP reduction after Ahmed valve surgery.^4^ In addition, IOP spikes after intravitreal injection of bevacizumab could be reduced through digital ocular massage.^5,6^ Patients can also perform ocular massage during acute angle closure attacks in order to lower their IOP before any medical treatment can be offered.^7^

IOP regulation is determined by the production and outflow of the aqueous humor. The majority of the aqueous humor drains through the trabecular meshwork (TM) into the lumen of the Schlemm’s canal, finally draining into aqueous veins and episcleral veins via collector channels, which is the conventional pathway.^8^ In addition, a small amount of aqueous humor is drained via uveoscleral outflow, which is the unconventional pathway.^9^

Kagemann et al.^10^ found that the SC area was significantly smaller in patients with glaucoma than in healthy subjects. Chung et al.^11^ recruited newly diagnosed open-angle glaucoma patients and measured their baseline SC area and TM width. Greater IOP reduction from IOP-lowering medications was found in patients with larger baseline SC areas. The TM width had no effect on IOP reduction. Although ocular massage has been advocated for due to its IOP lowering effects, the IOP reduction mechanism has not been clearly investigated. With anterior segment optical coherence tomography (AS-OCT), the corneoscleral region can be imaged with good resolution to identify the SC and TM. The current study investigated the effects of digital ocular massage on IOP variation. We hypothesized that IOP reduction through ocular massage was due to the dilation of the SC.

## Methods

Healthy adults were recruited from university campus. All participants had comprehensive eye examination including slit-lamp biomicroscopy and dilated fundus examination to rule out ocular diseases. The inclusion criteria were healthy ocular conditions and IOP of no greater than 21 mmHg. Participants with a history of ocular diseases were excluded. This study obtained ethical approval from the Institutional Review Board of The Hong Kong Polytechnic University (HSEARS2022022001) and was conducted according to the tenets of the Declaration of Helsinki. All participants provided informed consent prior to their participation in the study.

Baseline measurements included auto-refraction (Nidek ARK-510A, Nidek Co., Ltd., Japan) and rebound tonometry (Icare ic200, ICare Finland Oy, Helsinki, Finland). Corneoscleral region of the temporal side in the right eye was imaged using AS-OCT. The participants performed the ocular massage on their own. Rebound tonometry was performed immediately after the ocular massage, followed by AS-OCT of the same region. Rebound tonometry was performed 5 min after the ocular massage.

### Ocular massage

Each participant was taught to perform ocular massage using their fingertips.^12^ To standardize the procedure, a video clip regarding ocular massage was prepared and shown to each subject. The participants were instructed to close their eyes and place their index finger on the surface of the right upper eyelid. A light force was applied in circular motion for 2 s, followed by a 2-s release, and this 4-s cycle was repeated for 10 min. The participants were reminded not to open their eyes during the ocular massage. An examiner helped ensure that the participants performed the procedure correctly and for 10 min.

### Rebound tonometry

A rebound tonometer was used in order to obtain IOP immediately after ocular massage. This model showed good agreement with Goldmann applanation tonometry (GAT), in particular when GAT IOP ≤ 21mmHg^13^. Consequently, the acquisition of anterior chamber segment imaging could be achieved in a nearly instantaneous manner subsequent to ocular massage. The participants were advised to look straight ahead. The device was placed 4-8 mm from the center of the cornea and perpendicular to the surface. The device’s Series mode was used to automatically obtain six consecutive measurements after the first successful measurement. The device displayed an average value of the four best measurements. This IOP reading was considered the first attempt. Each acquisition consisted of two attempts, and the average results were used for the analysis. After a 5-min rest, a second acquisition consisting of two attempts was performed. These two acquisitions were used to calculate the test–retest variability (TRV). After performing the ocular massage, rebound tonometry was immediately performed. Five minutes after the ocular massage, a final acquisition was conducted.

### Imaging the corneoscleral region

**Figure 1:**
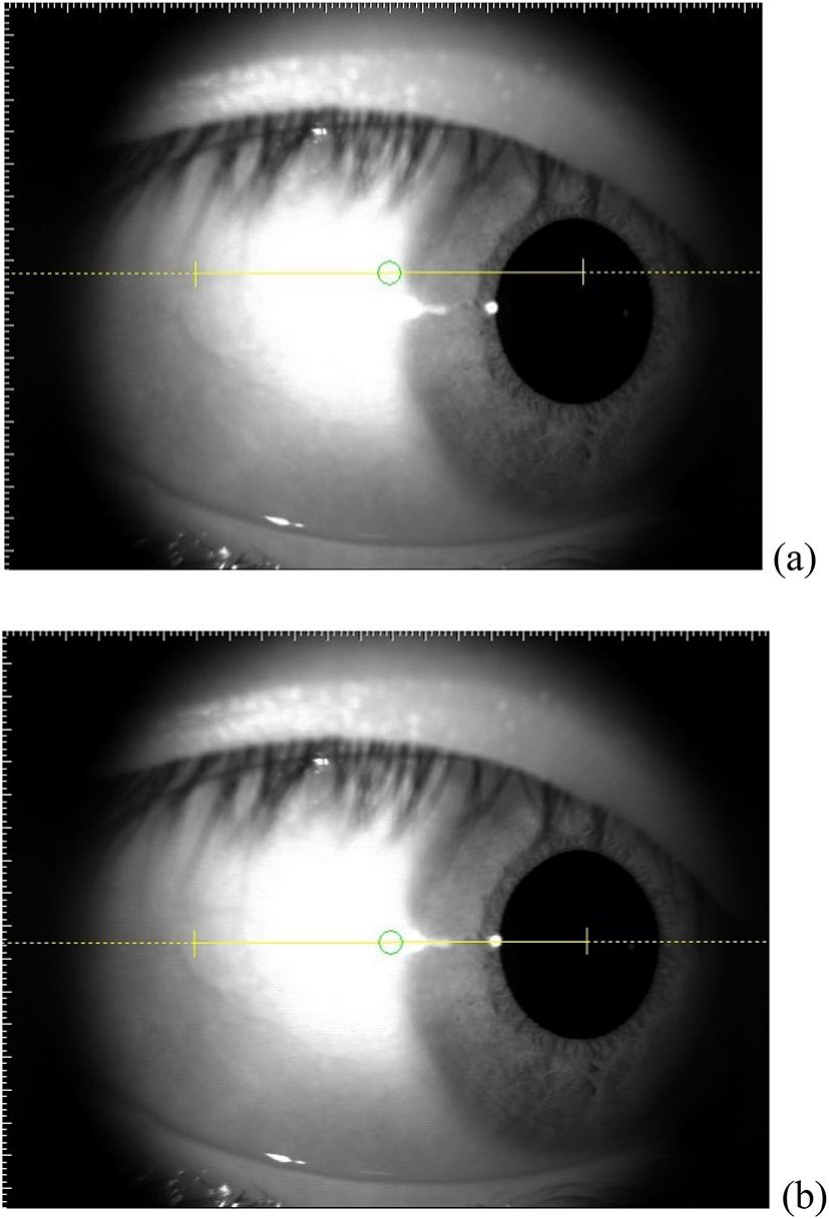
Right eye of one participant looking to the nasal side. (**a**) Yellow reference line above the corneal reflex. (**b**) Reference line aligned with the corneal reflex.

All participants underwent examination using swept-source AS-OCT (Casia SS-1000, Tomey Corporation, Nagoya, Japan). This device is designed for anterior segment imagining using a 1310 nm wavelength and a scan speed of 30,000 A-scans per second. A customized 3D-angle high-definition protocol was used (raster of 128 B-scans each with 512 A-scans over a 6 mm × 6 mm region). Participants were seated in a dark room and instructed to look at a nasal peripheral fixation light in order to acquire images of the temporal corneoscleral region of the right eye. To ensure consistency before and after the ocular massage, one examiner took the images and placed a reference line across the corneal reflex during each acquisition (Fig. 1).

**Figure 2:**
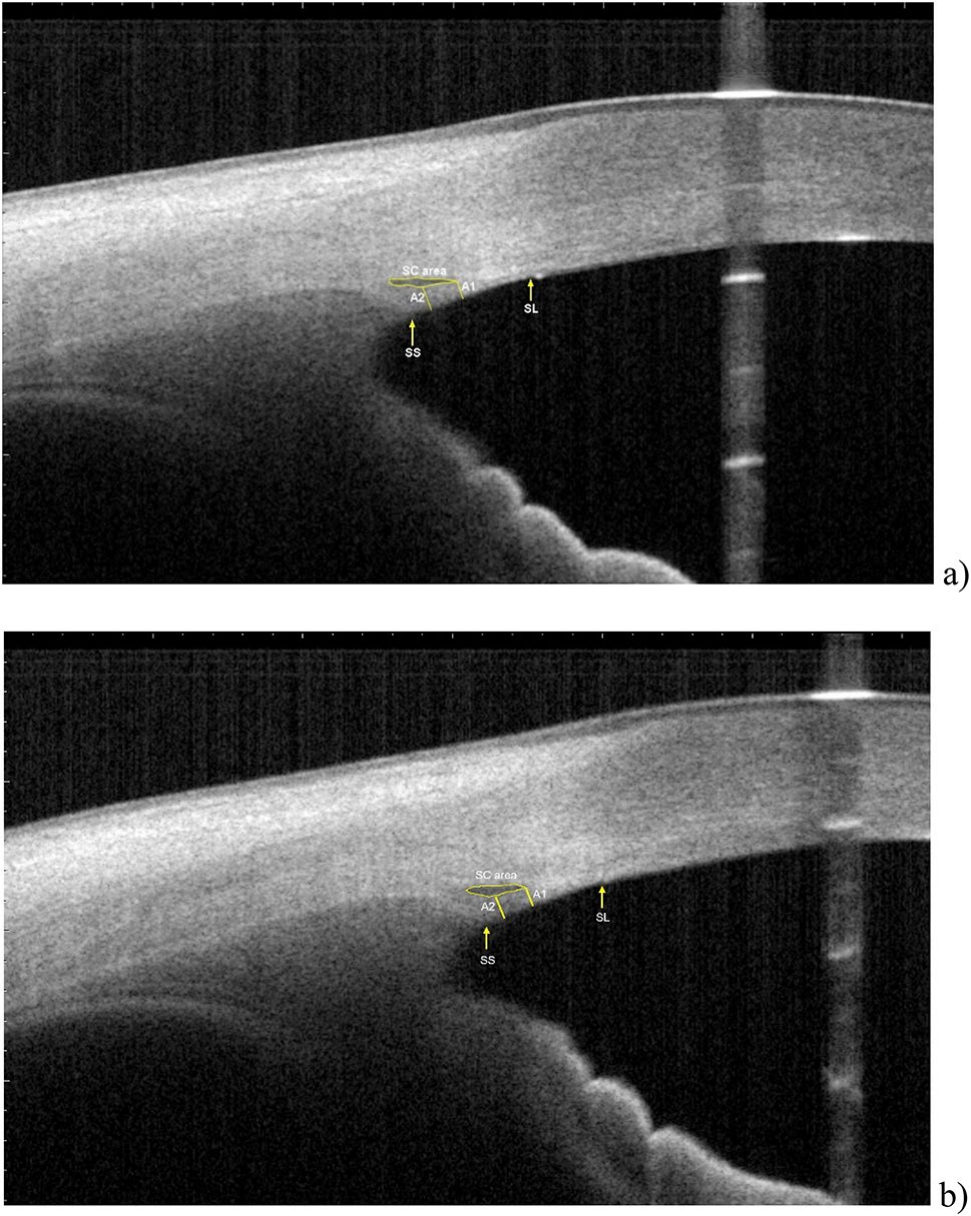
Measurements of Schlemm’s canal (SC) area, SC diameter, trabecular meshwork (TM) thickness, and TM width. SC was outlined by yellow line and measured by delineating the lumen. SC diameter is the distance from the anterior endpoint to the posterior endpoint of the SC. TM width was measured from the scleral spur (SS) to the Schwalbe line (SL). TM thickness was calculated as the average of two measurements, the perpendicular distances from the anterior endpoint (A1) and midpoint (A2) of SC to the inner layer of the TM. (**a**) Dimensions at baseline (subject 011): SC area = 18,035.4 μm^2^, SC diameter = 456.1 μm, TM width = 849.8 μm, TM thickness = 139.5 μm. (**b**) Dimensions immediately after ocular massage (subject 011): SC area = 23,030.4 μm^2^, SC length = 430.8 μm, TM width = 880.6 μm, TM thickness = 155.4 μm.

All captured images were exported and manually analyzed by an independent examiner using the ImageJ software (National Institutes of Health, USA). For each image, an outline of the SC was drawn freehand, and the area surrounded by the outline of the SC was depicted (Fig. 2). The diameter of the SC was measured from the posterior to the anterior end points of the outline. The TM width was defined as the distance between the scleral spur and Schwalbe’s line. The TM thickness was calculated as the average of two measurements made at the anterior end point of the SC and halfway down the SC.

### Statistical analysis

All statistical analyses were performed using GraphPad Prism (version 9.4.1, GraphPad Software, Boston, USA) and SPSS Statistics (version 26, IBM SPSS Inc., USA). In addition, the normality of the data was checked using the Shapiro–Wilk test. Normally distributed data were presented as mean ± standard deviation, or as median (interquartile range) if they were not normally distributed. The TRV of the rebound tonometry was calculated as 2.77× within-subject standard deviation from the first and second acquisitions before the ocular massage. Measurement of the SC area requires delineation of the SC boundary, which might have high variability. To study intra-examiner repeatability of SC area measurement, the first ten AS-OCT images obtained at baseline were measured again by the same examiner one week later. To study inter-examiner reproducibility, an independent examiner performed SC area measurements on the 10 images, and intraclass correlation coefficient (ICC) was used to assess reliability.^14^ ICC values between 0.61 and 0.80 were considered to represent substantial reliability, and ICC values exceeding 0.81 were considered as nearly perfect. The coefficient of variation (CoV) was calculated as the within-subject standard deviation divided by the mean of the measurements, the lower the CoV, the higher was the repeatability. Rebound tonometry before, immediately after, and 5 min after digital ocular massage was compared using the Friedman's test. Post-hoc tests with Bonferroni corrections were applied for pairwise comparisons. Corneoscleral parameters, namely SC area, SC diameter, TM width, and TM thickness, were compared before and after digital ocular massage using paired t-tests or Wilcoxon signed rank tests as appropriate. For corneoscleral parameters that showed significant changes after ocular massage, regression analysis was conducted with IOP changes. Assuming an average IOP of 15.6 mmHg and a 10% IOP change due to ocular massage in the normal population, at least 36 subjects were required to show the effect (0.05 alpha and 90% power). All tests were two-tailed. Statistical significance was set a *p* < 0.05.

## Results

Seven-five participants were initially recruited for the examination. Nine participants were excluded due to poor SC images that could not be identified. Sixty-six participants (33 males) were included (median age 22 years, range 18–40 years). The rebound tonometry TRV of the remaining subjects was 2.78 mmHg. The IOP of the two initial acquisitions exhibited similar results (first acquisition: 15.9 ± 2.6 mmHg; second acquisition: 15.6 ± 2.7 mmHg, paired *t*-test, *t* = 2.04, *p* = 0.05). The average value derived from these two acquisitions served as the baseline IOP prior to the application of ocular massage. Table 1 shows the IOP and corneoscleral parameters of the eligible subjects at baseline.

**Table 1:** Tonometry and corneoscleral parameters at baseline.

	Mean ± SD	Range
Rebound tonometry (mmHg)	15.7 ± 2.5	10.9–21.3
SC area (μm^2^)	10,063.5 ± 4165.6	2066.1–23,346.3
SC diameter (μm)	270.4 ± 84.1	78.8–498.7
TM width (μm)	733.3 ± 110.1	531.7–1013.2
TM thickness (μm)	149.8 (31.6)	79.1–308.9

Median (interquartile range) for data not normally distributed.

*SC* Schlemm’s canal, *TM* trabecular meshwork.

Table 2 shows the subjects’ IOP and corneoscleral parameters after ocular massage. All subjects demonstrated IOP reduction (Friedman’s test, *p* < 0.001). The mean IOP immediately after ocular massage was lower than at baseline, a mean reduction of −6.2 ± 1.9 mmHg. This effect persisted 5 min after the ocular massage, a mean reduction of −3.8 ± 2.0 mmHg. The post-hoc tests revealed significant difference in all pairwise comparisons (all *p* < 0.001).

**Table 2:** Tonometry and corneoscleral parameters after ocular massage.

	Mean ± SD	Range
Immediate IOP (mmHg)	9.6 ± 2.2*	5.5 to 16.4
Change in IOP (mmHg)	−6.2 ± 1.9	−3.1 to −12.1
5-min IOP (mmHg)	11.6 (4.5)*	7.6 to 19.9
Change in IOP (mmHg)	−3.8 ± 2.0	1.0 to −9.0
SC area (μm^2^)	10,151.0 (6149.6)*	3897.6 to 24,897.5
Change in SC area (μm^2^)	1217.1 ± 4096.8	−8665.25 to 10,381.72
SC diameter (μm)	276.5 ± 68.7	102.5 to 431.3
TM width (μm)	733.5 ± 111.6	523.4 to 1020.8
TM thickness (μm)	144.6 ± 25.3*	100.87 to 219.3
Change in TM thickness (μm)	−4.3 (29.1)	−158.1 to 54.0

Median (interquartile range) for data not normally distributed.

*SC* Schlemm’s canal, *TM* trabecular meshwork.

Difference from baseline: negative indicated measurement results small than baseline.

*Significant difference compared with baseline.

There was good reliability in measuring the SC area. The intra-examiner ICC and CoV values were 0.995 and 2.69%, respectively; while the inter-examiner ICC and CoV values were 0.973 and 6.91%, respectively. The SC area was significantly different from baseline after ocular massage (Wilcoxon signed rank test, *p* = 0.02). Since participants had a wide range of baseline SC area, from 3897.6 to 24,897.5 μm^2^, relative change of SC area in percentage was calculated for analysis. Twenty-four participants (36%) demonstrated collapse of the SC area (reduced from −2 to −52%) while the remaining participants showed expansion of the SC area (increased from 2 to 168%). The median change in SC area was 12% expansion. There were no significant changes in SC diameter (270.4 ± 84.1 μm vs 276.5 ± 68.7 μm, paired *t*-test, *p* = 0.499), and TM width (733.3 ± 110.1 μm vs 733.5 ± 111.6 μm, paired *t*-test, *p* = 0.988). TM thickness showed significant change (Wilcoxon signed rank test, *p* = 0.036).

**Figure 3:**
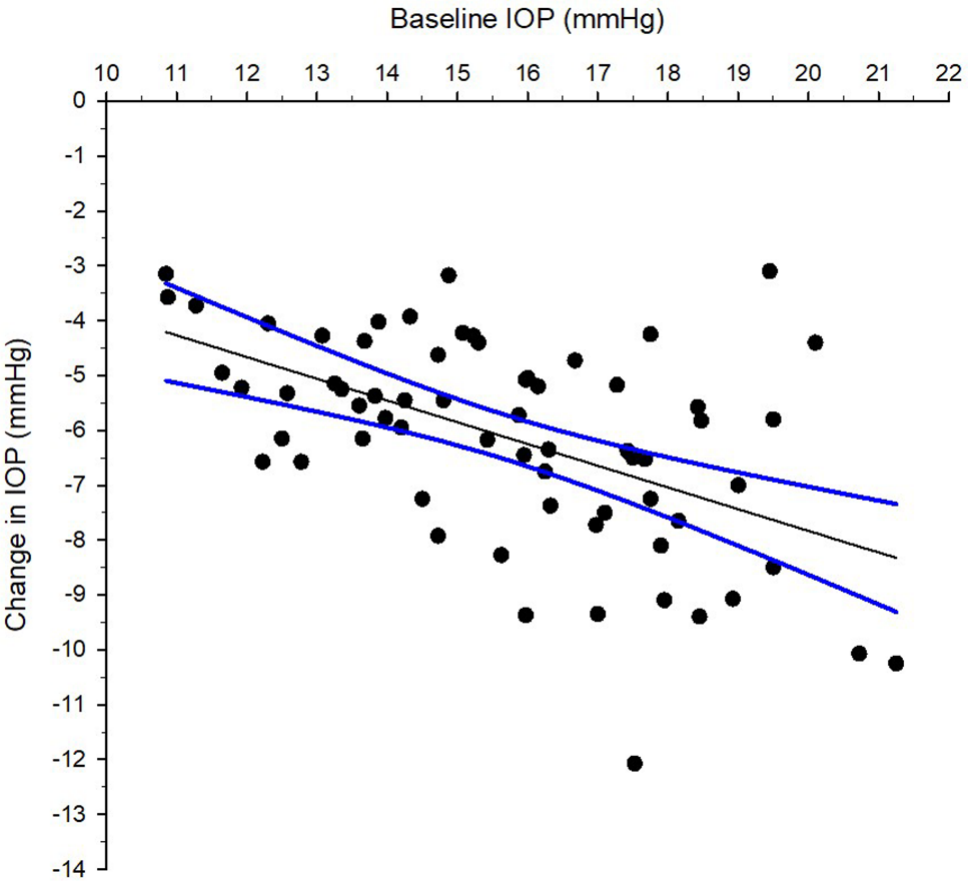
Correlation between intraocular pressure (IOP) change immediately after ocular massage with baseline IOP. Solid line is the regression line. Dotted lines are 95% confidence intervals. Pearson correlation coefficient r = −0.521, *p* < 0.001.

**Figure 4:**
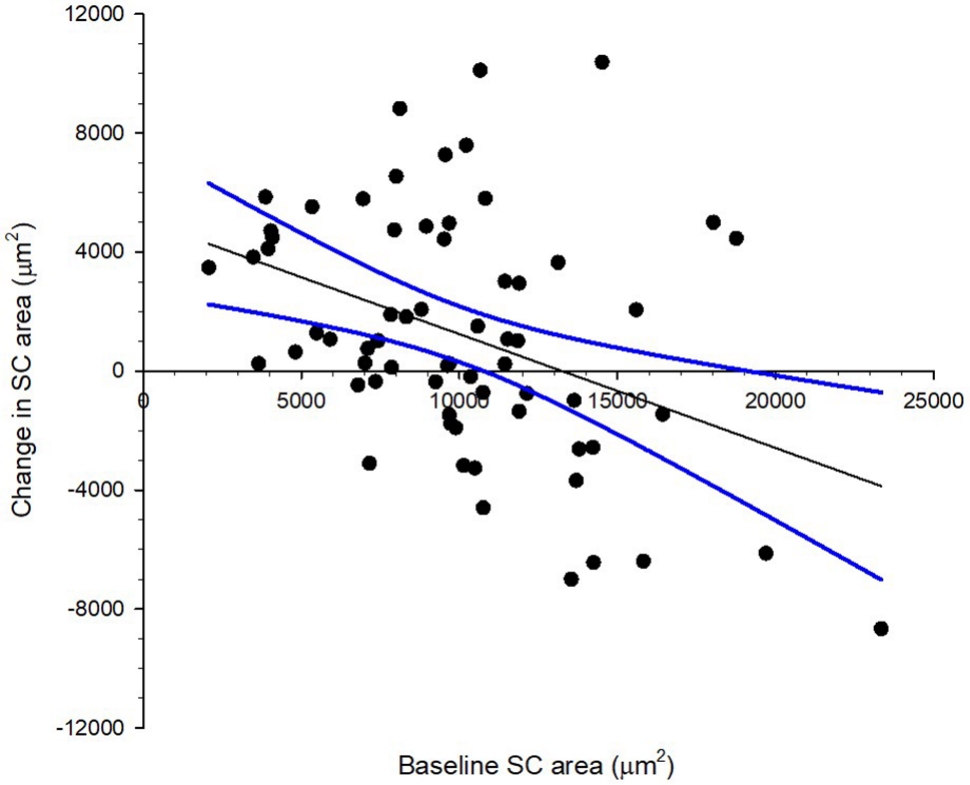
Correlation between Schlemm’s canal (SC) area change immediately after ocular massage with baseline SC area. Solid line is the regression line. Dotted lines are 95% confidence intervals. Pearson correlation coefficient r = −0.389, *p* < 0.001.

**Figure 5:**
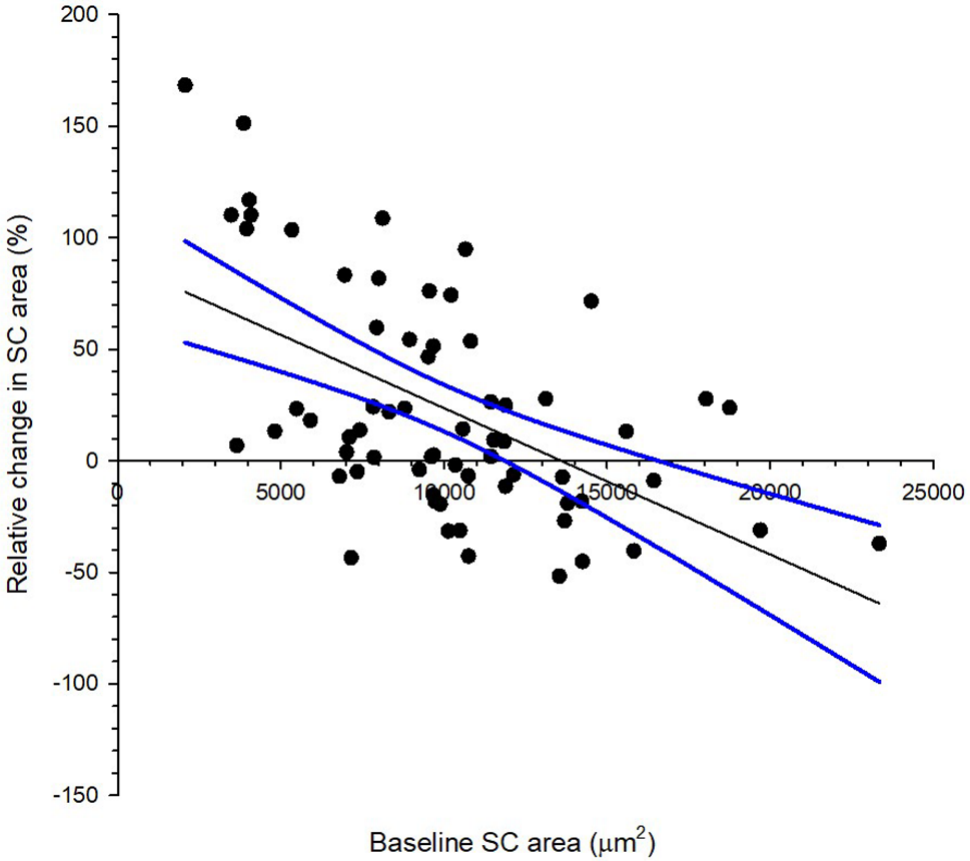
Correlation between relative change in Schlemm’s canal (SC) area immediately after ocular massage with baseline SC area. Solid line is the regression line. Dotted lines are 95% confidence intervals. Pearson correlation coefficient r = −0.544, *p* < 0.001.

**Figure 6:**
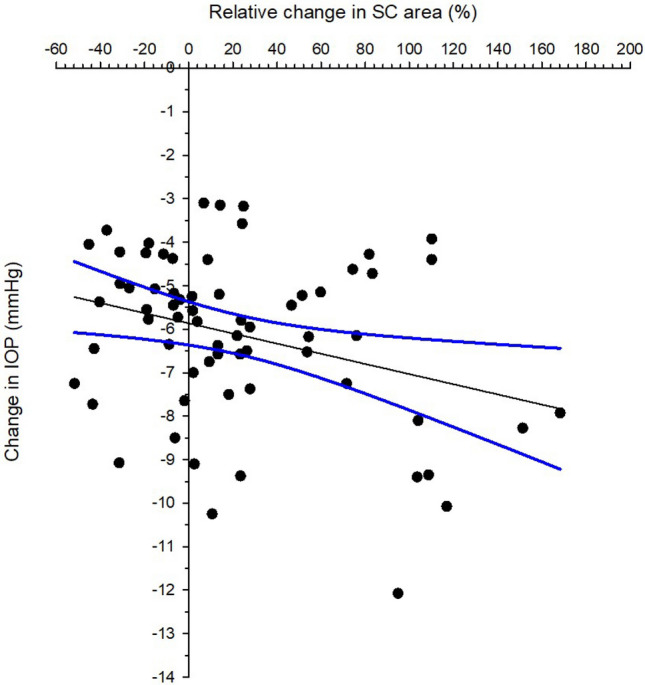
Correlation between intraocular pressure (IOP) change immediately after ocular massage and relative change in Schlemm’s canal. Solid line is the regression line. Dotted lines are 95% confidence intervals. Pearson correlation coefficient r = −0.306, *p* < 0.001.

IOP reduction was significant correlated with baseline IOP (Fig. 3, Pearson correlation coefficient r = −0.521, *p* < 0.001). Eyes with smaller baseline SC area demonstrated expansion and vice versa (Fig. 4, Pearson correlation coefficient r = −0.389, *p* < 0.001). Majority of the participants demonstrated SC area expansion which had smaller baseline SC area (Fig. 5, Pearson correlation coefficient r = −0.544, *p* < 0.001). There was significant association between IOP reduction and relative change of the SC area (Fig. 6, Pearson correlation coefficient r = −0.306, *p* < 0.001). Association between change in IOP and change in TM thickness was not significant (Spearman’s rho = 0.015, *p* = 0.902).

## Discussion

The current study confirmed the IOP lowering effect of digital ocular massage. For a TRV of 2.8 mmHg, all participants demonstrated IOP reductions from −3.1 to −12.1 mmHg immediately after ocular massage, which indicated true IOP reduction rather than any measurement error. This amount of change is equivalent to a 16% to 69% reduction from the baseline IOP. This effect was maintained for 5 min after ocular massage. Similar to the current results, McIlraith et al.^4^ revealed a 42% IOP reduction immediately after 10 s of digital ocular massage. Participants in their study were older with mean age of 59.5 years. In addition, Smith et al.^15^ studied the effect of digital ocular massage during the hypertensive phase after Ahmed valve surgery. In their study, digital ocular massage was performed by a doctor for 10 s, and the inclusion criterion was an IOP reduction of ≥ 20% at 1-h post-massage. This result would indicate a prolonged IOP lowering effect of ocular massage. In the current study, ocular massage was performed by the subjects for 10 min. The latest rebound tonometer model (ic200) was used owing to its good agreement with the GAT in particularly for IOP values within 21 mmHg.^16^ Regarding this, Nakakura et al.^17^ found that results from the Single mode and Series mode are interchangeable. However, the Series mode can speed up the measurement process for quicker evaluation of the SC and TM using AS-OCT. Given that GAT is the gold standard for measuring IOP, it is recommended that future studies should utilize GAT. An alternative to Goldmann applanation tonometry could be pneumatonometry. Bayraktar and Bayraktar^18^ (2005) demonstrated that IOPs derived from penumatonometry were relatively independent from central corneal thickness while GAT showed a significant correlation. However, IOP obtained from penumatonometry is normally higher than that from GAT and rebound tonometry (Bayraktar and Bayraktar^18^; Barkana and Gutfreund^19^). It is unknown if penumatonometry might find a larger difference in IOP after ocular massage. The findings of the current study demonstrate that ocular massage has a transient effect on reducing IOP, indicating that the duration of topical anaesthesia and application of fluorescent dye should have minimal impact of ocular massage on IOP reduction and alterations in Schlemm's canal.

IOP regulation depends on the aqueous humor dynamic. Considering that aqueous outflow occurs mainly via the TM and SC, a wider SC should facilitate better aqueous outflow to lower the IOP. Uveoscleral outflow is pressure independent.^20^ An increase or decrease in IOP at an initial phase of or stopping of the ocular massage, respectively, may have little effect on the uveoscleral outflow.^21^ Although the contribution of uveoscleral outflow might not be ignored, it is very difficult to measure it clinically. Unlike the uveoscleral pathway, outflow of aqueous humor through the TM is pressure dependent.^22^ To study this more, Allingham et al.^23^ studied autopsy eyes with high IOP glaucoma and found that these eyes had smaller SC areas, SC perimeters, and SC inner wall lengths than healthy eyes. In another study, Yuan et al.^24^ used a mathematical model to demonstrate increased outflow facility through dilation of the SC. Nowadays, with the introduction of spectral-domain OCT, the SC can be imaged in vivo.^25,26^ The Casia SS-1000 is a swept-source AS-OCT that makes the identification of the SC easier when compared with spectral-domain OCT owing to the deeper penetration ability of the former.^27^ Among 75 recruited subjects, the SC was difficult to identify in nine eyes (12%). This detection rate (88%) is similar to that reported by Usui et al.^28^ and slightly better than that reported by Gao et al.^29^, both of who utilized the Casia SS-1000. High-frequency ultrasound biomicroscopy provided a similar detection rate.^30^ In another study, Hong et al.^31^ employed spectral-domain OCT and found inter-examiner ICC and CoV of 0.80 and 13.4% for the SC area, respectively. Similar reliability values have been reported in adults^25,26^ and children population.^32^ In the current study, the first 10 AS-OCT images from 66 eligible subjects (15%) were selected to study the reliability of SC area measurements. Better ICC and CoV values were achieved, which could be due to the use of swept-source AS-OCT.

Among the four corneoscleral parameters studied to determine the contributing factors to lowering IOP through digital ocular massage, changes in the SC area and TM thickness were significantly associated with IOP reduction. Eyes with smaller baseline SC area had greater SC area expansion (Fig. 6). However, around one-third of the subjects showed collapse of the SC area after ocular massage, and their IOP reduction ranged from −3.7 to −9.0 mmHg. This was beyond the TRV of rebound tonometry, indicating a true IOP lowering effect. Although a significant association between IOP change and relative change of the SC area was observed, the correlation was weak (Pearson correlation coefficient r = −0.306). In addition, as only the temporal region was examined, we had no information regarding the changes that may have occurred in other corneoscleral regions as a result of the ocular massage. The SC contributes more to the IOP lowering phenomenon than the TM. In one study, Xiang et al.^33^ presented a −6 D accommodation stimulus to a group of children and monitored their SC and TM. They found that the SC area showed a 23% expansion and noted a 19% increase in the SC diameters. In contrast, the TM length increased by only 4% and the TM width did not change significantly. In another study, Chung et al.^11^ monitored both the nasal and temporal SC and TM in a group of newly diagnosed open-angle glaucoma patients before and after using IOP-lowering agents. The majority of the patients used prostaglandin analogues, while other patients used a carbonic anhydrase inhibitor/beta blocker fixed combination. These drugs mainly enhance aqueous outflow via the uveoscleral pathway or reduce aqueous production. Following their study, these researchers reported that greater baseline SC area was associated with higher IOP reduction, while the TM width was not significantly correlated with IOP reduction. In another study, Park et al.^34^ compared the SC and TM dimensions in treatment-naïve open-angle glaucoma patients before and after the use of IOP-lowering agents. Patients using prostaglandin analogues demonstrated an increase in TM thickness and a decrease in SC area. However, there was no change in the mean TM width. In addition, patients using a carbonic anhydrase inhibitor/beta blocker fixed combination did not show any changes in SC and TM dimensions. Notably, aqueous outflow resistance in the conventional pathway involves the connective tissue of the TM and inner wall of the SC.^35,36^ Although we found significant change in TM thickness, it did not contribute to IOP change (Spearman’s rho = 0.015, *p* = 0.902). However, the current study examined SC and TM dimensions rather than measuring aqueous outflow. Therefore, it cannot be concluded that stable TM dimensions imply no changes in outflow facility.

Only one corneoscleral region was observed in the current study. If both the nasal and temporal regions were measured, successful rebound tonometry after ocular massage would delay imaging of the corneoscleral region. In this study, the corneoscleral temporal region was examined, as lateral eye movements can affect IOP measurement. Kim et al.^37^ found a greater IOP increase with abduction (2.0 ± 2.7 mmHg) than with adduction (0.5 ± 2.9 mmHg) by employing rebound tonometry. Considering that rebound tonometry was conducted in the primary gaze, a smaller IOP rise during adduction in the right eye facilitated the evaluation of the temporal corneoscleral region. In a different study, Kagemann et al.^10^ found a larger SC area on the nasal side than on the temporal side. When using swept-source AS-OCT, Shi et al.^38^ found similar SC areas in these two regions. In addition, when Chen et al.^39^ used the Casia SS-1000, they found similar SC areas in two regions of high myopes. They also revealed a negative correlation between the SC area and IOP. Interestingly, the high myopia group had a larger SC area and diameter than the control group including low myopia, emmetropia, and low hyperopia. In contrast, the subjects in the current study had a wider range of ametropia, spherical equivalent of the right eye ranging from + 0.50 D to − 8.13D. However, the sample size was not large enough to further divide them into different refractive groups (only 18 eyes had myopia ≤ -6.00D).

Another phenomenon noted in this study was a trend of higher IOP reduction in eyes with greater expansion in the SC area (Fig. 6). Notably, the change in the SC diameter was not significant. A previous study found a negative correlation between IOP and SC area in both the temporal and nasal regions, however, the correlation with SC diameter also was not significant.^31^ The mode of AS-OCT acquisition allows for the evaluation of the SC in a two-dimensional manner. To better evaluate how the SC expands, 3-D imaging could help determine if the expansion is along the sagittal plane (horizontal), axial plane (vertical), or both.

This study had several limitations. First, IOP was measured by rebound tonometry rather than GAT. Although the ic200 model showed good agreement with GAT (95% LoA from −3 to 1 mmHg) when IOP was ≤ 21 mmHg, GAT should be utilized in future studies. Second, although all subjects watched the same video to learn how to perform digital ocular massage, there could still be variations in the force applied. However, while the use of educational videos can reduce variability and improve the effectiveness of ocular massage in general.^40,41^ Electronic eye massagers could be used to deliver a more stable massaging effect.^42^ Third, only the temporal corneoscleral region was measured in this study. If more regions were to be examined, morphological changes in corneoscleral parameters could be studied in more detail. There are no prior studies on anterior chamber dimensions to determine changes in the anterior chamber due to ocular massage. However, measuring more parameters could delay the evaluation of corneoscleral parameters after ocular massage. Fourth, the sample size was small and was limited to healthy young Chinese individuals. A post hoc power analysis was performed based on changes in the SC area, and there was 84% power to detect true changes in the SC area after ocular massage. However, the current results may not be the same for other ethnic groups owing to the presence of different anterior chamber dimensions.^43^ It is also worth conducting a similar study in older adults, as scleral stiffness is age-dependent.^44^ The IOP response from ocular massage could be different. Additionally, it would be useful to know when would IOP return to its baseline level. Corneal biomechanical properties were not measured. Any alteration of corneal biomechanics could affect IOP measurements. Our previous study using electronic eye massager did not find big effect on corneal hysteresis and corneal resistance factor.^42^ Liu et al. found reduction of corneal hysteresis and corneal resistance factor from eye rubbing.^45^ Subjects were instructed to rub their eyes as they usually would in case of itchiness, not ocular massage as adopted in the current study. Eye rubbing intensity among participants could also be very different. Li et al. applied a corneal visualization Scheimpflug technology (Corvis ST) to monitor corneal biomechanics before and after 1-min of eye rubbing. Eye rubbing appears to make the cornea softer.^46^ Future studies should also monitor corneal biomechanics from digital ocular massage. The contralateral eye that was not massaged could serve as self-control. Unfortunately, the contralateral eyes were not measured in the current study.

## Conclusions

Simple digital ocular massage is an effective way to lower IOP on a temporary basis. The SC area change was positively associated with IOP reduction. Additional research is necessary to investigate whether similar effects can occur in glaucoma patients or in the elderly.

## Data Availability

The datasets used and/or analysed during the current study available from the corresponding author on reasonable request.
